# 777 Improving Parent Communication With Family Rounds In The Pediatric Critical Care Unit (PICU)

**DOI:** 10.1093/jbcr/irac012.330

**Published:** 2022-03-23

**Authors:** Olivia Arredondo, Mary Day, Sally A Martens, Kathleen S Romanowski, Soman Sen, Tina L Palmieri, David G Greenhalgh

**Affiliations:** Shriners Hospital for Children Northern California, Sacramento, California; Shriners Hospital for Children Northern California, Sacramento, California; Shriners Hospital for Children Northern California, Sacramento, California; University of California, Davis and Shriners Hospitals for California Northern California, Sacramento, California; UC Davis, Sacramento, California; UC Davis and Shriners Hospitals for Children Northern California, Sacramento, California; University of California - Davis, Sacramento, California

## Abstract

**Introduction:**

Effective communication between pediatric burn patients, their family members and the health care team is crucial to developing a "working alliance" and improving their overall experience in the Pediatric Intensive Care Unit (PICU). The aim of the project is to standardize and improve patient and parent communication through the implementation of weekly inter-disciplinary family rounds.

**Methods:**

Our nursing team developed the innovate PICU survey which is a 9 question Likert scale survey that evaluates patient and family satisfaction. All pediatric patients being discharged or transferred from the PICU received the PICU survey. After reviewing baseline data, communication between patient/parents and the health care team was identified as a potential targeted area for improving satisfaction scores. Aimed at improving communication, we established weekly inter-disciplinary family rounds. Each family was designated a specific day of the week for inter-disciplinary family rounds and staff provided families with a pre-printed standardized flyer explaining the process and were encouraged to write down questions for discussion. Survey scores from burn patients who received inter-disciplinary family rounds were compared to baseline scores, as well as to the scores of other pediatric patients of services that do not utilize family rounds.

**Results:**

Prior to implementing family rounds, our PICU survey average score was 4.9/5 out of 6 surveys. After implementing inter-disciplinary family rounds, our average score for pediatric burn patients was 4.9/5 out of 10 surveys. The average scores of pediatric patients of services that did not utilize family rounds 4.7/5 out of 19 surveys.

**Conclusions:**

Scheduled inter-disciplinary family rounds can improve communication and over patient care satisfaction in pediatric patients with complex critical care issues related to burn injuries. Scheduled family rounds may also be beneficial for other non-burn pediatric ICU patients.

